# Survival of swine pathogens in compost formed from preprocessed carcasses

**DOI:** 10.1111/tbed.13876

**Published:** 2020-10-23

**Authors:** Brent Pepin, Todd Williams, Dale Polson, Phillip Gauger, Scott Dee

**Affiliations:** ^1^ Pipestone Veterinary Services Pipestone MN USA; ^2^ Boehringer Ingelheim Animal Health Duluth GA USA; ^3^ Iowa State University Ames IA USA

**Keywords:** compost, grinding, pathogen, PEDV, pre‐processing, PRRSV

## Abstract

An introduction of a Foreign Animal Disease (FAD) like African Swine Fever Virus (ASF) would be financially devastating. For example, ASF, a highly contagious pathogen with high mortality rates, is a World Health Organization reportable disease that has recently been spreading across Asia and Europe. Control of ASF would likely require mass euthanasia of infected and exposed animals similar to the United Kingdom’s elimination of Foot and Mouth Disease (FMD). Subsequent disposal of infectious carcasses must adequately eliminate the virus and prevent further transmission of the disease. Although composting swine carcasses is widely used throughout the industry, limited data is available describing pathogen survival or elimination during this process. While current methods have evaluated the composting of swine carcasses under temperature‐controlled settings, they have not considered the effects of adverse weather conditions (e.g., cold winter conditions) where composting is routinely performed. This study utilized preprocessing (grinding) of swine carcasses prior to composting, which decreases the amount of required carbon material and land space. The ability of composting to reduce the level of viral nucleic acid during cold weather conditions and the risk of environmental contamination that may occur during preprocessing was evaluated. In this study, pigs challenged with Porcine Reproductive and Respiratory Syndrome Virus (PRRSV) and Porcine Epidemic Diarrhea Virus (PEDV), common domestic diseases, before euthanasia provided infectious carcasses containing pathogen surrogates. Composting of preprocessed carcasses achieved adequate temperatures necessary to eliminate FAD and common swine pathogens during cold weather conditions (monitored by compost temperature over time, virus diagnostic testing, and swine bioassay for PRRSV and PEDV). Under the conditions of this study, composting preprocessed carcasses presents minimal risk to air and groundwater contamination. In conclusion, composting preprocessed euthanized swine under adverse weather conditions is a safe and feasible option for mass disposal of infected carcasses.

## 1 INTRODUCTION

The diagnosis of a swine Foreign Animal Disease (FAD) in the United States (US) has a projected loss of $2.6‒$50 billion to the pork industry (Carriquiry et al., 2020; Dee et al., 2018; Paarlberg et al., 2002, 2009). To reduce the financial burden, quick and effective disease containment and elimination methods are necessary. The diagnosis of a FAD in the US will evoke a “stamping out policy” implementing depopulation of all confirmed and exposed swine to eliminate the outbreak (World Organization for Animal Health, 2019). Disease elimination through depopulation requires a method of mass disposal of infected carcasses using approved methods that properly eliminate viable virus for on‐site disease control and prevention of site‐to‐site transmission (Costa & Akdeniz, 2019). Studies demonstrating environmental risk and virus reduction capabilities of carcass disposal methods are limited, including common viruses in the swine industry such as Porcine Reproductive and Respiratory Syndrome Virus (PRRSV) and Porcine Epidemic Diarrhea Virus (PEDV). The known airborne transmission capabilities of PRRSV and the environmental survivability of up to 9 months for PEDV make these pathogens immediate domestic concerns for mass disposal situations (Arruda et al., 2019; Li et al., 2018).

Outbreaks of Foot and mouth disease (FMD) in the United Kingdom have highlighted the limitations of current mass disposal methods (Wilkinson, 2007). On‐farm carcass disposal using mass burial is restricted based on groundwater levels, burning presents both public perception and human safety concerns, and the use of rendering facilities and landfills is troublesome from a biosecurity standpoint due to the requirement of transporting infectious carcasses off‐site (Costa & Akdeniz, 2019; Scudamore et al., 2002; Wilkinson, 2007). However, composting is recognized as an environmentally safe method of carcass disposal for routine and emergency use in Australia, New Zealand, the US, and Canada (Guan et al., 2010; Wilkinson, 2007).

Traditional composting that involves covering unaltered carcasses with a carbon source is commonplace for swine mortality (Costa & Akdeniz, 2019; Erickson et al., 2004; Kalbasi‐Ashtari et al., 2005; Rynk, 2003). However, previously described methods of preprocessing carcasses (grinding carcasses into small particle sizes through mechanical crushing) before compost pile formation may be better suited for mass depopulation (Erickson et al., 2004; Kalbasi‐Ashtari et al., 2005). The benefits of preprocessing carcasses include lower amounts of carbon material and faster tissue degradation using less land space than traditional composting methods that use un‐processed swine carcasses (Erickson et al., 2004; Kalbasi‐Ashtari et al., 2005; Rynk, 2003). When preprocessing, it is recommended to compost equal volumes of carcass and the carbon material (biomass) for greater efficiency of carcass degradation. However, most studies of preprocessed carcasses involve cattle with limited information available for swine (Erickson et al., 2004; Kalbasi‐Ashtari et al., 2005; Rynk, 2003). In addition, the majority of swine compost studies are conducted in temperature‐controlled containers or buildings that do not consider adverse environmental effects that may occur in open‐air outdoor settings where mass composting is conducted (Glanville et al., 2016; Guan et al., 2010; Kalbasi‐Ashtari et al., 2005; Vitosh‐Sillman et al., 2017). The ability to compost outdoors and achieve the required high temperatures is of particular concern in cold weather conditions during winter in the Midwestern US, where the majority of the country’s pork production exists (Oppedahl, 2020). The risk of environmental contamination and the spread of viruses is another significant concern during mass carcass disposal for which limited information is available (Costa & Akdeniz, 2019). Therefore, this study’s objectives were to analyze potential risks of environmental contamination of preprocessed infectious swine carcasses, the ability to achieve required compost temperatures to eliminate viable swine pathogens under cold weather conditions, and assess the ability of preprocessed compost material to reduce or eliminate PRRSV and PEDV viability.

## 2 MATERIALS AND METHODS

### 2.1 Animals

Animals were used under the guidelines and approval of the Pipestone Research Institutional Animal Care and Use Committee (IACUC) protocol ID# 2020‐003. All animals were humanely euthanized by penetrating captive bolt method and confirmed insensible, according to the American Veterinary Medical Association Euthanasia Guidelines (Leary et al., 2020).

### 2.2 Viruses

Due to the safety risk of using FAD like ASF, common domestic swine viral pathogens must be used. The well documented airborne and aerosolization properties of PRRSV and the environmental survival capabilities of PEDV make these pathogens good models for assessing environmental contamination risk and compost elimination (Arruda et al., 2019; Li et al., 2018). Five hundred sixty‐six (566), 18‐23 kg pigs 8 weeks in age were challenged with PRRSV and PEDV in an Animal Biosafety level 2 (ABSL2) research facility by an oral transmission route in the feed (Dee et al., 2020). The concentration of challenge material for PRRSV and PEDV was 1 × 10^5^ 50% tissue culture infectious dose per ml (TCID_50_/ml) for each pathogen. Pigs were confirmed exposed via oral fluid PCR and infected by serum and fecal swab PCR for PRRSV and PEDV, respectively. Forty (40) carcasses of 132 kg pigs 6 months in age from a local processing plant were also utilized for preprocessing in this study. The bioassay contained 30 pigs housed in an ABSL2 research facility and challenged with their respective infected tissue homogenate.

### 2.3 Pre‐processing (grinding)

Swine carcasses were preprocessed prior to composting by grinding equal volumes of carcass and biomass using a 750 hp horizontal grinder (Rotochopper® FP‐66 B‐series, Rotochopper, INC, St. Martin, MN). The processed pig and biomass blended material was formed into windrows that consisted of long, narrow compost piles with a large exposed surface area for passive aeration (Erickson et al., 2004).

### 2.4 Compost biomass

Three different types of carbon source biomass were used and compared: woodchips, cornstalk bales, and a 1:1 combination of each source. A separate windrow was formed for each of the biomass types. Pig carcasses in each specific windrow biomass type were preprocessed with their respective carbon biomass.

### 2.5 Windrow formation

**FIGURE 1:**
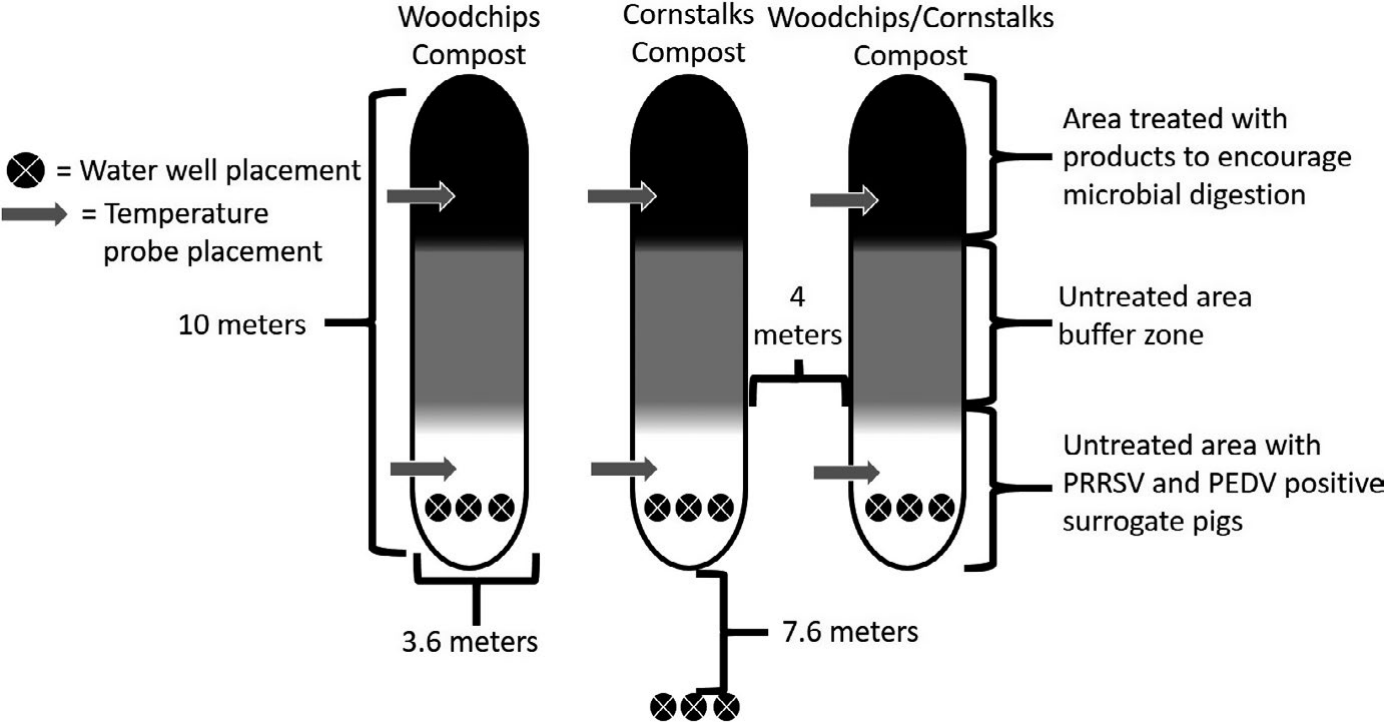
Compost windrow dimensions with water well layout, designated microbial digestion application areas, virus surrogate pig locations, and temperature probe positions

Three compost windrows were formed, each representing one of the biomass materials (woodchips, cornstalks, and a 1:1 combination of the two). A base layer of each biomass type was applied first, followed by a layer of the preprocessed carcass material combined with the biomass. Finally, the preprocessed carcass material was covered with a layer of the carbon material of each windrow’s specific biomass type. The end section of each windrow contained only preprocessed carcasses of pigs confirmed infected with PRRSV and PEDV (approximately 544 kg of carcass weight per windrow). The remaining portion of each windrow was composed of approximately 5600 kg carcass per windrow. Final dimensions consisted of three separate windrows at 3.6m wide, 10m long, and 2.1 m high (Figure 1).

### 2.6 Water well location and water sample collection

Three water wells were located under the end section of each biomass type that contained the infected surrogate pigs (Figure 1). The wells were constructed at the depths of 0.15 m (6 inches), 0.46 m (18 inches), and 0.91 m (36 inches) below the ground surface. Three wells at the same depths were also placed 7.6 m (25 feet) downhill from the compost windrows. Wells were drilled to their respective depths, and 0.15 m of slotted polyvinyl chloride (PVC) approximately 0.102 m wide was placed at the bottom of each well. The slotted PVC was connected to enough solid PVC pipe to sit above the ground to remain accessible after windrow formation was complete. Sodium bentonite was applied to the area around the top of the well where it extended above the ground. Between collections, the wells were capped to prevent extraneous water introduction from sources that would not represent groundwater. Water samples were collected using a 3‐way catheter valve with sufficient rubber tubbing to reach the bottom of each well. A 60cc plastic syringe created sufficient negative pressure to collect a water sample from the respective well. The first well water collection occurred at day five post windrow formation and continued once weekly until the completion of the study.

### 2.7 Aerosol sampling

Aerosol collection occurred with the use of six air collectors with 200 L per minute flow (Innovaprep, Drexel, MO). Collectors were positioned downwind from the horizontal grinder and were operated during the entirety of the preprocessing procedure. Two air collectors were placed at each distance of 46 m (50 yards), 91 m (100 yards), and 137 m (150 yards) from the horizontal grinder. The air collection occurred for 3 hr, beginning at the start of the carcass preprocessing. The preprocessing was completed at approximately 2.5 hr, but the aerosol collection at each distance ran for the full 3 hr. An aerosol sample was collected from each device after every 60 min run time (18 total samples). Samples for testing were collected at the end of the one‐hour runtime by filter 0.075% Tween 20/PBS wet‐foam elution kits (Innovaprep, Drexel, MO).

### 2.8 Compost treatment

The windrow section opposite of the known PRRSV and PEDV positive surrogate pig carcasses was treated with products that promote microbial digestion. The windrow section between the viral surrogate pigs and the microbial digestion treated windrow section acted as a buffer between the two areas. In the treated segment of each windrow, three products were applied via a hand pump sprayer in equal volumes that are traditionally used to encourage liquid manure digestion. The application was over the processed carcass material before the top layer of biomass was added to complete the windrow. The three products included the digestive microbe blends Pit Accelerator (ProfitPro, LLC, Albert Lea, MN), Microbial Manure Master™ (ProfitPro, LCC, Albert Lea, MN) and the liquid biocatalyst Eubio‐NBS (Eubio Tec, Albert Lea, MN).

### 2.9 Compost sample collection

An initial sample was collected after the preprocessing was complete from each biomass type from the virus surrogate carcass section, immediately behind the location of the water wells. Two compost samples were taken at each following collection by a careful deconstruction of the top biomass cover layer of the pile until the processed material was reached. A minimum of 10 g of the processed compost material was collected from the shallow depth of processed material. Another 10 g sample was then collected from at least 0.91 m deep into the processed material through further careful deconstruction of the processed compost material. Samples were collected wearing a shoulder‐length plastic obstetrician sleeve, changing sleeves between each grab sample and windrow type. Each 10 g sample was placed into a sterile Whirl‐Pak bag (Nasco, Fort Atkinson, WI, US) and stored at −80°C until testing. Compost samples were collected daily for the first five days after windrow formation, followed by weekly sample collection. Weekly compost sampling continued until two consecutive PCR negative results were obtained from each windrow.

### 2.10 Compost grab sample processing

Compost grab samples were processed for testing as described in a previous study where 10 g of compost material is combined with 25 ml of minimal essential media (Vitosh‐Sillman et al., 2017). The compost MEM mixture was blended in a stomacher (Stomacher^®^ 400 Circulator, Seward Limited, Worthing, West Sussex, UK) for 2 min at 230 rpm, and the supernatant separated for diagnostic testing.

### 2.11 Serum and fecal Sample Collection

Blood collection for serum occurred via jugular venipuncture with a single‐use collection system. Blood was centrifuged at 1,800 ×*g* for 10 min, and serum separated for testing. Fecal swabs were collected with individual sterile swabs (FisherFinest® Transport Swab, Fisher Scientific, Pittsburgh, PA) by introduction into the rectum.

### 2.12 Polymerase chain reaction testing

PRRSV and PEDV reverse transcription real‐time polymerase chain reaction (PCR) diagnostic testing were performed according to laboratory standard diagnostic procedures in which a cycle threshold of ≥ 38 was considered negative for virus nucleic acid detection. Standard laboratory diagnostic procedures for oral fluid PCR was used for testing compost, water, and air collection elution samples for PRRSV and PEDV. All PCR positive processed compost samples were saved in a −80°C freezer for bioassay testing. All PRRSV and PEDV diagnostics were completed at the Animal Disease Research and Diagnostic Laboratory at South Dakota State University according to standard diagnostic procedures.

A commercially available porcine (*Sus scrofa*) DNA real‐time PCR kit was used according to manufacturer directions to test for the presence of swine DNA on air and compost samples (RapidFinder™ Pork ID Kit, ThermoFisher Scientific, Waltham, MA, USA). Testing occurred on all air elution samples. Only compost samples that tested negative by PRRSV and PEDV PCR from day 0–5 post windrow formation were tested. In weeks 2 and 3, a compost sample from each biomass type was collected at the 0.91m depth for swine DNA testing in addition to the normal compost grab samples. Swine DNA PCR testing was performed at the Iowa State University Veterinary Diagnostic Laboratory.

### 2.13 Compost temperature and weather monitoring

This study targeted starting in February in the upper Midwest when winter conditions in Minnesota average negative 11°C (−11°C) to assess cold weather effects on the preprocessing and compost method (https://arcgis.dnr.state.mn.us/ewr/climatetrends/#). A local research weather monitoring station gathered the daily high and low temperatures during the study, including the daily precipitation and snowfall (https://swroc.cfans.umn.edu/weather). Compost temperatures were monitored with 0.91 m long industrial compost thermometer probes (Reotemp Instruments Corp., San Diego, CA) placed at two locations in each pile, one in the area treated with products to encourage microbial digestion and one in the untreated compost area in the site of the known surrogate positive pigs. Compost temperatures were collected daily for the first five days following windrow formation and then weekly to the study’s completion.

### 2.14 Bioassay

The PRRSV and PEDV PCR positive compost samples were evaluated for infectivity via swine bioassay. Thirty (30) pigs approximately 3 weeks in age and negative for PRRSV and PEDV by PCR on serum and fecal testing, respectively, were used. Pigs were housed in an ABSL2 research facility and randomly allocated into ten pens with three pigs/pen. Pen design prevented any physical or manure contact between the treatment groups. Challenge material consisted of PCR positive compost samples. On day 0, pigs were administered 2 ml intramuscularly and 2 ml orally with a specific PCR positive compost sample that was processed as described prior to PCR testing. Pigs were tested following exposure on day 3 and 7 post‐challenge via individual serum and fecal swab by PRRSV and PEDV PCR, respectively. Pigs were monitored daily for clinical signs of diarrhea post‐challenge. After the bioassay, all pigs were humanely euthanized.

## 3 RESULTS

### 3.1 Compost temperature by treatment and biomass type

Regardless of biomass type or microbial digestion treatment, similar temperature peaks were achieved (Table 1). By day 3 post‐windrow formation (pwf), each biomass type reached an internal temperature of 60°C or higher and maintained that temperature for at least 48 hr except for the treated section of the combination biomass windrow that decreased to 55.6°C on day 5 pwf. Woodchips biomass reached the highest peak temperature at 75.6°C on day 3 pwf. Both sections of woodchips and the untreated section of the cornstalks reached temperatures >60°C by day 2 pwf, a day before the combination biomass. The mean peak air temperature for the first 5 days pwf, during the same time of the peak windrow temperatures, was 3°C, and the mean low air temperature was −5.1°C. Extraneous compost moisture from the weather during the first 5 days pwf was minimal with 0.05cm of rainfall and 0.51cm of snowfall.

**TABLE 1:** Weather conditions^†^ and temperature readings of compost by biomass type^‡^ and treatment^§^

	Time post windrow formation				Woodchips		Cornstalks		Combination	
	Total Rainfall (cm)	Total Snowfall (cm)	High (°C)	Low (°C)	Treated (°C)	Un‐treated (°C)	Treated (°C)	Un‐treated (°C)	Treated (°C)	Un‐treated (°C)
Day 0	0.05	0.51	−5.0	−10.6	7.8	7.8	23.3	18.9	11.1	11.1
Day 1	0.00	0.00	−1.1	−6.7	15.6	15.6	26.7	46.1	12.2	14.4
Day 2	0.00	0.00	6.7	−3.3	**66.7** ^¶^	**65.6**	30.0	**65.6**	55.6	43.3
Day 3	0.00	0.00	7.2	−3.3	**75.6**	**74.4**	**63.3**	**67.8**	**68.9**	**71.1**
Day 4	0.00	0.00	3.3	−3.3	**70.0**	**70.0**	**63.3**	**64.4**	**60.0**	**60.0**
Day 5	0.00	0.00	6.7	−3.3	**73.9**	**72.2**	**62.2**	**62.2**	55.6	**66.7**
Week 2	0.46	2.03	10.0	−8.3	7.2	7.2	55.6	56.7	37.8	**60.0**
Week 3	1.52	0.76	6.7	−11.1	37.8	10.0	27.8	14.4	11.1	48.9
Week 4	0.76	0.51	10.6	−11.1	11.1	10.0	25.6	12.2	8.9	8.9
Week 5	5.66	0.76	15.6	−6.7	32.2	28.9	18.9	34.4	15.6	20.0

^†^ For day 0–5 pwf, the total rainfall and snowfall are recorded as the total for that day. For weeks 2–5, the rain and snowfall are recorded as the total for the week. High and low temperatures are reported daily for day 0–5 pwf, and the high and low temperatures are reported for the entire week for weeks 2–5 pwf.

^‡^ Three biomass types used for windrow formation include woodchips, cornstalks, and a combination of half woodchips and half cornstalks.

^§^ Each compost carbon source type had a section applied with microbial digestion stimulating treatment (Treated) and a section that was not applied with the microbial product (Untreated).

^¶^ Bolded values indicate temperature readings ≥ 60°C which is documented high enough to inactivate ASF with 20 min of exposure.

### 3.2 Aerosol sample analysis

All filter elution samples tested negative for PRRSV and PEDV by PCR. Wind speed during aerosol collection averaged 6.4‐8kmh (4–5 miles per hour).

### 3.3 Water sample analysis

Eleven total water well samples were able to be attained throughout the study (Table 2). The only PCR positive water sample was collected at week 5 from the 0.15 m well located beneath the cornstalks biomass. Water samples were successfully collected at 0.15 m and 0.46 m depths. However, sample collection was not successful at the 0.91 m depth.

**TABLE 2:** Reverse transcription, real‐time PRRSV and PEDV PCR cycle threshold values detected in water samples collected from wells placed at 15 cm, 46 cm, and 91 cm beneath their respective compost biomass types^†^ and wells placed downhill at the same depths of their respective compost biomass types

Week of collection after compost pile formation and virus tested		Woodchips			Cornstalks			Combination			Downhill^‡^		
		15cm	46cm	91cm	15cm	46cm	91cm	15cm	46cm	91cm	15cm	46cm	91cm
Week 1	PEDV	‒^§^	‒	‒	‒	‒	‒	neg	‒	‒	neg	‒	‒
	PRRSV	‒	‒	‒	‒	‒	‒	neg	‒	‒	neg	‒	‒
Week 2	PEDV	neg^¶^	‒	‒	‒	‒	‒	neg	‒	‒	‒	‒	‒
	PRRSV	neg	‒	‒	‒	‒	‒	neg	‒	‒	‒	‒	‒
Week 3	PEDV	neg	‒	‒	‒	‒	‒	‒	‒	‒	‒	‒	‒
	PRRSV	neg	‒	‒	‒	‒	‒	‒	‒	‒	‒	‒	‒
Week 4	PEDV	neg	‒	‒	‒	‒	‒	‒	‒	‒	‒	neg	‒
	PRRSV	neg	‒	‒	‒	‒	‒	‒	‒	‒	‒	neg	‒
Week 5	PEDV	‒	‒	‒	**35.18**	‒	‒	neg	‒	‒	‒	neg	‒
	PRRSV	‒	‒	‒	neg	‒	‒	neg	‒	‒	‒	neg	‒
Week 6	PEDV	‒	‒	‒	‒	‒	‒	‒	‒	‒	‒	neg	‒
	PRRSV	‒	‒	‒	‒	‒	‒	‒	‒	‒	‒	neg	‒

^†^ Three carbon source types used for windrow compost formation include woodchips, cornstalks, and a combination of half woodchips and half cornstalks.

^‡^ Wells placed 7.6m downhill from the three compost windrows.

^§^ “‒” signifies water collection was attempted but no sample present at that collection period.

^¶^ “neg” signifies a negative PCR test result on the sample that collected for the well.

### 3.4 Compost biomass analysis

At day 0 pwf (day carcass preprocessing and windrow formation occurred), all 3 biomass type windrows were PRRSV and PEDV PCR positive (Table 3). Woodchips had a greater number of PRRSV and PEDV PCR positive samples compared to cornstalk biomass and the combination biomass material. Cornstalks biomass demonstrated the least number of PRRSV and PEDV positive samples. The final PRRSV PCR positive result was detected on day 4 pwf in the woodchip biomass. The final PEDV PCR positive was detected at week 2 in both the woodchip and combination biomass samples.

**TABLE 3:** Reverse transcription real‐time PCR cycle threshold values for PED and PRRSV detected in woodchip, cornstalk, or combination compost samples collected at 0–5 days and 2–4 weeks post windrow formation

Day post windrow formation and virus tested		Woodchips^†^		Cornstalks		Combination	
Day 0	PEDV	29.60		29.03		27.85	
	PRRSV	29.07		27.76		26.90	
		**Deep** ^‡^	**Shallow**	**Deep**	**Shallow**	**Deep**	**Shallow**
Day 1	PEDV	34.69	‒^§^	33.19	NS^¶^	‒	‒
	PRRSV	35.13	‒	28.51	NS	‒	‒
Day 2	PEDV	34.2	‒	‒	‒	‒	‒
	PRRSV	34.15	‒	‒	‒	‒	‒
Day 3	PEDV	‒	35.59	‒	‒	‒	‒
	PRRSV	‒	‒	‒	‒	‒	‒
Day 4	PEDV	‒	34.32	‒	‒	‒	‒
	PRRSV	‒	27.12	‒	‒	‒	‒
Day 5	PEDV	‒	‒	‒	‒	‒	‒
	PRRSV	‒	‒	‒	‒	‒	‒
Week 2	PEDV	37.05	‒	‒	‒	‒	33.8
	PRRSV	‒	‒	‒	‒	‒	‒
Week 3	PEDV	‒	‒	‒	‒	‒	‒
	PRRSV	‒	‒	‒	‒	‒	‒
Week 4	PEDV	‒	‒	‒	‒	‒	‒
	PRRSV	‒	‒	‒	‒	‒	‒

^†^ Three carbon source types used for windrow formation include woodchips, cornstalks, and a combination of half woodchips and half cornstalks.

^‡^ Deep compost sample was at least 0.91 m into the preprocessed carcass material of the compost. Shallow samples collected from the outer section of the preprocessed carcass material directly under the carbon source covering of windrow.

^§^ “NS” denotes a sample collected on which PCR could not be performed.

^¶^ “‒” represents a PCR negative sample.

### 3.5 Swine DNA analysis

Swine DNA was detected in the sample of each biomass type up to day 5 pwf (Table 4). Swine DNA was also detected at weeks 2 and 3 pwf from each biomass type. Swine DNA was also detected in the aerosol samples collected at hour 3 in one sample, each at the 46 m and the 91 m distances (Table 5). No swine DNA was detected at the 137 m distance.

**TABLE 4:** Reverse transcription real‐time PCR cycle threshold values detecting swine DNA in compost samples by biomass type^†^

Time post windrow formation	Woodchips		Cornstalks		Combination	
	Deep^‡^	Shallow	Deep	Shallow	Deep	Shallow
Day 1	‒^§^	neg^¶^	‒	‒	22.3	33.0
Day 2	‒	‒	21.9	13.5	21.4	21.8
Day 3	23.0	‒	19.3	18.5	21.9	29.0
Day 4	20.5	‒	13.5	19.1	24.2	27.1
Day 5	25.0	26.4	20.8	21.7	34.5	23.2
Week 2	23.7		19.4		neg	
Week 3	34.3		neg		34.1	

^†^ Three compost carbon source types used for windrow formation include woodchips, cornstalks, and a combination of half woodchips and half cornstalks.

^‡^ Deep compost sample was at least 0.91 m into the preprocessed carcass material of the compost. Shallow samples collected from the outer section of the preprocessed carcass material directly under the carbon source covering of windrow.

^§^ “‒” denotes the swine DNA PCR was not performed on that sample.

^¶^ “neg” signifies a negative PCR test result on the sample tested.

**TABLE 5:** Reverse transcription real‐time PCR cycle threshold values detecting swine DNA in air samples collected during carcass preprocessing

Distance from grinder and hour of collection^†^	**Air collector 1**	**Air collector 2**
46 m 1 hr	Undetected^‡^	Undetected
46 m 2 hr	Undetected	Undetected
46 m 3 hr	Undetected	34.7
	**Air collector 3**	**Air collector 4**
91 m 1 hr	Undetected	Undetected
91 m 2 hr	Undetected	Undetected
91 m 3 hr	33.6	Undetected
	**Air collector 5**	**Air collector 6**
137 m 1 hr	Undetected	Undetected
137 m 2 hr	Undetected	Undetected
137 m 3 hr	Undetected	Undetected

^†^ Aerosol collectors were operating at 200 L/minute flow rate at 1 hr run times for each sample. Six total collectors were used with two at each given distance from the horizontal grinder during the carcass preprocessing.

### 3.6 Bioassay

Pigs administered the PRRSV PCR positive compost samples remained negative for PRRSV nucleic acid throughout the study. Woodchips were the only biomass to provide PEDV PCR positive rectal swabs in combination with clinical diarrhea consistent with PEDV infection (Table 6). Treatment groups challenged with PEDV PCR positive compost samples from week 2 pwf, did not produce any clinical signs in the challenged pigs and all pigs tested negative by PEDV PCR on day 7 post‐challenge. One treatment group from the combination biomass had one pig die from unrelated illness before the first diagnostic sample collection (Table 6).

**TABLE 6:** Individual PEDV reverse transcription real‐time PCR positive fecal swabs collected from pigs administered PEDV PCR positive biomass type and day post windrow formation

Positive sample from day post windrow formation	Woodchips Biomass Carbon Source		
	Day 4 Post‐challenge	Day 7 Post‐ Challenge	^†^Clinical signs:
Day 0	3/3	‒^‡^	Yes
Day 1	0/3	0	‒
Day 2	0/3	2/3	Yes
Day 3	1/3	0/3	‒
Day 4	3/3	‒	Yes
Week 2	1/3	0/3	‒

Positive sample from day post windrow formation	**Cornstalks biomass carbon source**		
	Day 4 Post‐challenge	Day 7 Post‐ Challenge	Clinical signs:
Day 0	0/3	0/3	‒
Day 1	0/3	0/3	‒

Positive sample from day post windrow formation	**50% Woodchips/ 50% Cornstalks Biomass Carbon Source**		
	Day 4 Post‐challenge	Day 7 Post‐ Challenge	Clinical signs:
Day 0	0/3	0/3	‒
Week 2	1/2^§^	0/2	‒

^†^ Documents if pigs had clinical signs of diarrhea consistent with PEDV infection.

^‡^ If all 3 piglets tested positive for PEDV and showed clinical signs they were removed from the study.

^§^ One pig died before the first sample collection.

## 4 DISCUSSION

The study objectives included analyzing, under cold weather conditions, the potential risks of environmental contamination of preprocessed infectious swine carcasses, the ability to achieve required compost temperatures to eliminate viable swine pathogens, and assess the ability of preprocessed compost material to denature PRRSV and PEDV using a swine bioassay. Given the common use of composting in the swine industry, we would expect the processing of carcasses before composting to achieve adequate temperatures for pathogen elimination, including for FAD viruses such as ASF. Preprocessing of carcasses for compost, even under cold weather conditions, appears to be an effective method for PRRSV and PEDV nucleic acid reduction. Regardless of the biomass type used, windrows achieved adequate temperatures for viral pathogen elimination, reaching temperatures that are reported to be able to denature ASF (Mazur‐Panasuik et al., 2019; United States Department of Agriculture, 2018). However, this study shows that compost material can remain infectious for a period of time after windrow formation, which had not been previously demonstrated. Under the conditions of the study, aerosolization of viruses and groundwater contamination from preprocessing and composting of swine carcasses appears minimal. Previous studies have not utilized the detection of porcine DNA on aerosolization or compost samples over time for comparison.

The process of preprocessing prior to composting has not been evaluated for potential risks of environmental contamination with viruses or swine material, even though the generation of leachate is a concern (Costa & Akdeniz, 2019). Although this study did not demonstrate the absence of environmental contamination, the results suggest that the risk of aerosolization of viral RNA and swine DNA is low during the carcass preprocessing. PCR positive viral RNA was not detected in the filter elution samples, although swine DNA was detected only at the 46 m and the 91 m distances but not 137 m, which validates the aerosol sampling method was adequate. The air collectors were placed downwind from the horizontal grinder, allowing the best location for the collection of virus and swine nucleic acid. However, a limitation of this study is that air samples were only collected out to 137 m from the preprocessing procedure suggesting further pathogen aerosolization studies are needed during carcass preprocessing.

The biomass that was placed underneath the preprocessed carcass compost material may have prevented significant leaching of virus nucleic acid into the groundwater during this study, although windrows that lacked the base layer were not included in the study for comparison. In a study completed on an above‐ground burial with Seneca Valley Virus PCR positive pigs, carcasses were placed directly within the surrounding soil and leaching of the virus nucleic acid down to a depth of 46 cm, but not 91 cm was detected (B. Thaler, personal communication, April 22, 2020). In locations or time periods of more considerable rain or snowfall, the potential for leaching of pathogens into the soil and groundwater may increase (Chatterjee et al., 2013; Grisey et al., 2010). Considering this study was performed during the winter months, the frozen soil under the windrows may have reduced or prevented potential pathogen leaching under the conditions of the study (Gupta et al., 2004; Jamieson et al., 2002). Further research is needed to assess the potential leaching of swine viruses to the groundwater from contaminated carcasses.

In addition to reaching adequate temperatures, this study was designed to assess the ability of composting preprocessed carcasses in cold weather conditions to reduce pathogen viability to mimic a similar situation during a FAD outbreak. Studies have shown that composting can eliminate viable swine viruses such as FMD and PEDV; however, many of these studies were performed under controlled and enclosed environmental conditions (Costa & Akdeniz, 2019; Guan et al., 2010; Vitosh‐Sillman et al., 2017). The current study evaluated similar parameters under natural conditions that occur during outdoor winter weather situations. In the windrow sections treated with microbial stimulants, the most considerable numerical differences were observed in the combination biomass windrow. However, overall treated and untreated sections of the windrows were numerically similar in compost temperatures. According to the US Environmental Protection Agency, pathogen reduction by time‐temperature is classified as Class A or Class B, which is used to assess virus elimination capability (Costa & Akdeniz, 2019). Class A qualification requires composting temperatures to reach 55°C for three consecutive days (Costa & Akdeniz, 2019). A limitation of this study was compost temperatures were not recorded frequently enough to confirm continual pile temperature. However, Table 1 shows all three biomasses did reach a temperature > 55°C for at least 3 consecutive days’ showing potential for Class A classification. Specifically pertaining to ASF, maintaining 60°C for 15–20 min is documented as adequate to eliminate the virus, which all compost types would have achieved under the conditions of this study (Mazur‐Panasuik et al., 2019; United States Department of Agriculture, 2018). In addition, the preprocessing compost biomass was able to reach temperatures reported to eliminate pathogens even under the adverse cold weather conditions (Table 1).

Although widely used as a standard method for carcass disposal worldwide, there are limited reports that evaluate virus survival in composted swine carcasses (Christensen et al., 2002; Wilkinson, 2007). Temperature is an important parameter for the inactivation of pathogens; however, temperature alone does not account for the heterogeneous composition of the compost. This heterogeneous composition creates microbiological environments and internal temperatures within the windrow that can vary (Christensen et al., 2002). Due to the heterogeneous microbial environments that affect pathogen survival, the monitoring of indicator organisms along with temperatures is recommended (Christensen et al., 2002). Differences in microbial environments that can be monitored include the outer layers and inner layers of the compost material where outer layers are expected to be cooler compared to the internal, deeper areas, potentially affecting pathogen detection over time (Wilkinson, 2007). In the current study, compost sampling was targeted at a shallow outer location of the carcass/biomass blended material and the deeper internal region (minimum 0.91 m into the carcass/biomass blended material) to provide a comparison of two microbial environments. Prior to complete windrow formation, each biomass type after processing was confirmed PCR positive for PRRSV and PEDV as indicator surrogate viruses. The current study did not show a noticeable difference in the detection of PRRSV and PEDV nucleic acid between the shallow or deep sampling locations (Table 3). The preprocessing of carcasses before composting creates smaller particles, which will increase the efficiency of heat inactivation compared to whole carcasses, which may explain why deep and shallow compost samples did not represent an apparent difference in nucleic acid detection (Wilkinson, 2007). Studies looking at the pathogen survival of PEDV and Pseudorabies Virus in compost demonstrated that neither virus was detected after 36 and 29 days, respectively, but neither tested the compost material during the immediate days and weeks after formation as in the current study (Morrow et al., 1995; Vitosh‐Sillman et al., 2017). The current study is in agreement with previous studies that composting can successfully eliminate swine viral pathogens but demonstrates the potential infectivity of compost material the first weeks after windrow formation (Table 3). The potential infectivity of the detected nucleic acid is confirmed by the bioassay results (Table 6). ASF is known to be a heat‐sensitive pathogen estimated to require exposure to temperatures 56–60°C for rapid inactivation (Mazur‐Panasuik et al., 2019). In traditional compost with full swine carcasses, it is estimated to require 40 hr of exposure at 60°C for the center of the carcass to reach 56°C (Franke‐Whittle & Insam, 2013). The bone marrow of ASF infected pigs is known to contain high loads of virus; therefore, reaching high internal temperatures is critical (Gale, 2004). The preprocessing of carcasses eliminates the discrepancy between the internal carcass and surface temperatures. This due to the small particle size created by the grinding and mechanical crushing of the carcasses, removing the internal carcass sub‐environment. This suggests that the preprocessing of carcasses, as described in the current study, will inactivate heat‐sensitive pathogens like ASF with greater efficiency than traditional compost.

ASF survival in different matrices was found to be dependent on the moisture content, with the most persistence in drier materials like straw and hay (Mazur‐Panasuik & Wozniakowski, 2020). Two limitations of the current study include no evaluation of compost moisture content or carbon: nitrogen ratios. However, woodchips used for compost can have a moisture content of 14.4% or lower, whereas cornstalks can have moisture content up to 76% (Ima & Mann, 2007; Tannous, 2015; Xu et al., 2020). The lower moisture content of the woodchips may have contributed to the extended detection of viral nucleic acid by PCR compared to windrows containing cornstalks. This is consistent with the bioassay results where PEDV PCR positive rectal swabs were detected from pigs administered the PEDV PCR positive woodchip biomass (Table 6). This study suggests that cornstalks may be a better compost material than woodchips for the reduction or potential elimination of viable swine viruses in the first 2 weeks pwf.

The pathogens utilized in the current study are both RNA viruses, whereas swine nucleic acid is double‐stranded DNA. Prior studies have demonstrated that double‐stranded DNA is more stable compared to RNA (Grosjean, 2009). The difference in the stability of RNA and DNA may explain the longer detection of swine DNA that was observed in the compost material compared to viral RNA. The expected larger quantity of DNA from the swine carcass compared to the viral nucleic acid may have also contributed to the extended detection. To the knowledge of the authors, this is the first study that evaluated the detection of swine DNA in compost material over time. These data suggest detectable swine DNA decreased over time based on elevated *Ct* values observed in all biomass types. However, the expected length of time porcine DNA could be detected from composted carcasses remains unknown.

The use of bioassay to determine the infectious capability of a detected virus is a highly sensitive and conclusive method (Zimmerman et al., 2012). However, the use of bioassay to determine the infectious ability of compost material in swine has not been well documented. The results of the current study demonstrate that PRRSV infectivity in compost material is short‐lived regardless of the biomass material. However, PEDV nucleic acid detected in the compost material remained infectious in the woodchips out to 4 days after windrow formation but appeared non‐infectious at the later time points. The current study data suggests that PEDV viability may be reduced in compost material that contains cornstalk biomass. A single pig was PEDV PCR positive in feces at day 4 post‐challenge in the groups administered the woodchip and combination biomass collected at week 2. However, no clinical signs were observed, and the individual fecal swabs in the same pigs were negative on PCR three days later. This suggests the PEDV detected at that time was not genuinely infectious, or the concentration of infectious material was low enough that replication was short‐lived.

The current study suggests the ability of composting to reduce the viability of swine viruses and, therefore, may indicate potential success with a FAD. To confirm this similar studies using viruses such as ASF are needed. However, the current study also indicates that different biomass material may influence the ability to decrease viable virus over time. Woodchips are commonly used as biomass for compost. However, this study demonstrates that woodchips may be less effective at reducing viral nucleic acid or virus viability than other biomass material. In a mass depopulation event where multiple swine sites must dispose of large numbers of carcasses, the availability of carbon sources for compost biomass is a concern. Therefore, various options for compost material may be important when a rapid response is needed to eliminate the transmission of disease, particularly a FAD. The advantage of preprocessing carcasses is that a smaller footprint (available area or land) is required compared to traditional non‐processed carcass composting. Preprocessing carcasses before composting also allows for adequate temperatures to be achieved in a short period of time. The current study demonstrates preprocessing may remove the need to turn the windrow to achieve an additional peak in compost temperatures to render pathogens non‐viable, as commonly done in traditional composting. This study also suggests that the risk of environmental contamination from carcass preprocessing is minimal, but requires further evaluation. Current research on the effect of composting, the compost biomass types used, and moisture content on pathogen survival in swine is limited and more research in this area is warranted.

## 5 CONFLICT OF INTEREST

The authors had no conflicts of interest in the completion and reported results of this study.

## ETHICAL APPROVAL

The authors confirm that the ethical policies of the journal have been followed. All procedures involving animals were in accordance with the Pipestone Research IACUC, protocol ID#: 2020–003.

## Data Availability

The data that support the findings of this study are available from the corresponding author upon reasonable request.
